# Robotic Appendiceal Ureteroplasty for Complex Right-sided Ureteral Strictures: Technical Approaches and Midterm Outcomes

**DOI:** 10.1016/j.euros.2026.05.018

**Published:** 2026-07-07

**Authors:** Kunlin Yang, Xinfei Li, Liqin Xu, Yixuan Huang, Hongjian Zhu, Zhihua Li, Liqun Zhou, Xuesong Li

**Affiliations:** a Department of Urology, Peking University First Hospital, Beijing, China; b Institute of Urology, Peking University, Beijing, China; c National Urological Cancer Center, Beijing, China; d Beijing Jian Gong Hospital, Beijing, China

**Keywords:** Ureteral stricture, Robotic surgery, Appendiceal ureteroplasty

## Abstract

**Background:**

Reconstruction of complex ureteral strictures remains challenging. The appendix provides an autologous alternative for ureteral reconstruction, distinguished from oral mucosal grafts by its intrinsic vascularity.

**Objective:**

To describe robotic appendiceal ureteroplasty (RAU) techniques and evaluate their midterm outcomes.

**Design, setting, and participants:**

From May 2019 to September 2024, 31 patients with right-sided complex ureteral strictures underwent RAU at a single institution. All procedures were performed using a robotic single-docking approach. Perioperative and follow-up data were prospectively collected.

**Intervention:**

Robotic appendiceal ureteroplasty (RAU) using four different surgical techniques (single-docking approach), depending on stricture location and length.

**Outcome measurements and statistical analysis:**

Outcomes included operative time, estimated blood loss, length of hospital stay, perioperative complications, and overall success rate (symptom relief and radiographic patency). Descriptive statistics were used; success rate is reported with 95% confidence interval.

**Results and limitations:**

Ureteral strictures were located between the ureteropelvic junction and mid-ureter in 29 patients (93.5%). Mean (range) stricture length was 5.5 (3–25) cm. Mean operative time was 176.4 (99–265) min, estimated blood loss was 42.7 (10–200) ml, and postoperative hospitalization was 4.5 (3–12) days. No open conversions or major perioperative complications occurred. Median (range) follow-up was 28 (12–67) months. The overall success rate was 100% (30/30; 95% CI 88.4%–100%). Limitations include the single-center design, lack of a control group, and limited sample size.

**Conclusions:**

RAU is a safe and effective approach for managing complex right-sided ureteral strictures; however, careful patient selection is essential, taking into account both the characteristics of the stricture and the condition of the appendix.

## Take Home Message

Robotic appendiceal ureteroplasty using onlay flap, interposition, and replacement techniques offers a versatile and effective solution for complex right-sided ureteral strictures with favorable perioperative outcomes. This approach can avoid the need for oral mucosa ureteroplasty or ileal ureter replacement.

ADVANCING PRACTICE **What does this study add?**

Robotic appendiceal ureteroplasty represents a resourceful utilization of vascularized autologous tissue for complex ureteral reconstruction. The proposed algorithm for technique selection provides surgeons with a reproducible strategy to optimize reconstruction while preserving native urinary tract function.

**Clinical Relevance**

Complex ureteral strictures remain challenging when tension-free ureteroureterostomy is not possible. For right-sided strictures, the appendix offers a vascularized autologous reconstructive option that may be used either as a detubularized onlay flap or as a tubularized substitute, depending on the length, location, and degree of ureteral obliteration. Compared with ileal ureteral replacement, appendiceal reconstruction may avoid bowel anastomosis and limit bowel-related morbidity; compared with oral mucosal graft ureteroplasty, it provides intrinsic vascularity. However, its use depends on appendiceal length, quality, lumen caliber, and absence of inflammation or prior appendectomy. Surgeons should therefore prepare alternative strategies, such as oral mucosal graft ureteroplasty or ileal ureteral replacement, when appendiceal reconstruction is not feasible. Associate Editor: Véronique Phé.

**Patient Summary**

This study reports the largest series of robotic appendiceal ureteroplasty to date. Using the appendix to repair complex right-sided ureteral strictures appears safe and effective.

## 1 Introduction

Reconstruction of complex ureteral strictures remains a surgical challenge. When tension-free end-to-end anastomosis is not feasible, the use of autologous tissue for ureteral reconstruction becomes an essential and effective alternative. Various tissues, including oral mucosa [1], appendix [2], ileum [3], and colon [4], have been successfully utilized for this purpose. Oral mucosal graft (OMG) ureteroplasty has recently attracted considerable attention as a reliable option for both left- and right-sided ureteral strictures, with favorable outcomes reported by multiple centers [1], [5]. For right-sided strictures, the appendix offers an additional reconstructive alternative, distinguished by its intrinsic and well-preserved vascular supply [2], [6], [7], [8].

Ureteral substitution using the appendix was first reported by Melnikoff in 1912 [9]. In 1971, Wesolowski described the use of appendiceal replacement for a solitary right kidney with a proximal stricture [10]. In 1976, Weinberg reported the use of the appendix for repairing a schistosomiasis-related long ureteral stricture with clinical success [11]. However, end-to-end ureteroappendiceal anastomosis by open or laparoscopic approaches is challenging and may carry a significant risk of stricture recurrence [12].

To overcome the aforementioned risk, the detubularized appendiceal flap was anastomosed to the posterior ureteral plate of the longitudinally incised ureter. In 2009, Reggio et al. [13] reported the first laparoscopic appendiceal onlay flap ureteroplasty with subjective and objective success rates of 66% and 100%, respectively. To date, some case reports and small case series on appendiceal onlay flaps, interposition, and replacement have been published [6], [7], [8], [12], [14].

However, ensuring successful appendiceal ureteroplasty (AU) requires careful attention to numerous technical details. Robotic surgery is precise and may help us achieve better outcomes than prior approaches. However, existing reports still lack systematic descriptions of such approaches. This study aims to provide standardized approaches for the appropriate application of these techniques and to report the midterm outcomes of robotic AU (RAU) for the treatment of complex ureteral strictures.

## 2 Patients and methods

A total of 31 patients were enrolled from May 2019 to September 2024. The affected side in all patients was the right side. The indications for surgery were a ureteral stricture not amenable to direct anastomosis, no history of appendectomy, and no inflammation in the appendix. For all patients, when AU was not feasible, alternative options included OMG ureteroplasty or ileal ureteral replacement (IUR), and preoperative preparation was the same as previously reported [3], [5]. All surgeries were performed by the same surgical team using robotic Si or Xi systems.

All patient demographics and perioperative results were prospectively collected from our Reconstruction of Urinary Tract: Technology, Epidemiology and Result database. A *p* value <0.05 was considered to indicate statistical significance.

### 2.1 Surgical technique

**Fig. 1:**
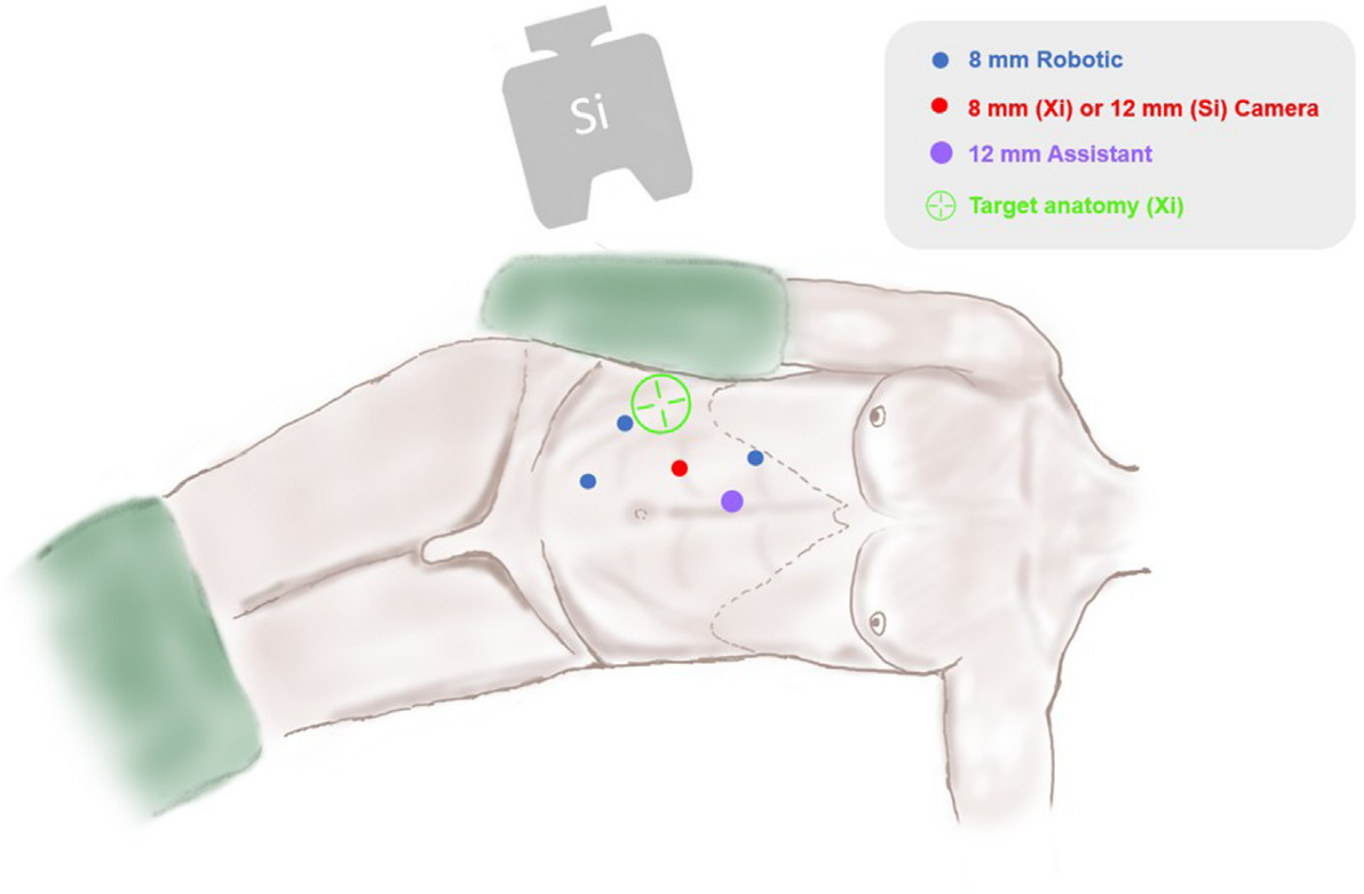
Patient position, port placement, and robot docking for single-docking robotic appendiceal ureteroplasty. Blue circle, 8 mm robotic port; red circle, camera port, 8 mm robotic port (DaVinci Xi system) or 12 mm port (Da Vinci Si system); and purple circle, 12 mm assistant port. The target anatomy (green cross) of Da Vinci Xi system is located between the ureteral stricture site and the cecum.

The patient was placed in a left lateral position (45–60°), and the port placement was similar to that in our previous report (Fig. 1) [3]. All procedures were performed using a transperitoneal approach with a single-docking technique (Fig. 1). The procedure started with mobilization of the ascending colon and the ileocecal region to the midline, followed by dissection of the ureteral stricture segment. Fibrotic scar tissue was excised as needed, with careful preservation of the periureteral vascularity. The choice of reconstruction depended on the luminal patency and suitability of the incised segment as the posterior wall. Afterwards, the suitability of the appendix for ureteral reconstruction was assessed, with the specific details outlined below.

#### 2.1.1 Appendiceal flap

The appendix was longitudinally spatulated along the antimesenteric border and used as a detubularized onlay flap to enlarge the ureteral lumen [7].

##### 2.1.1.1 Simple appendiceal flap

**Fig. 2:**
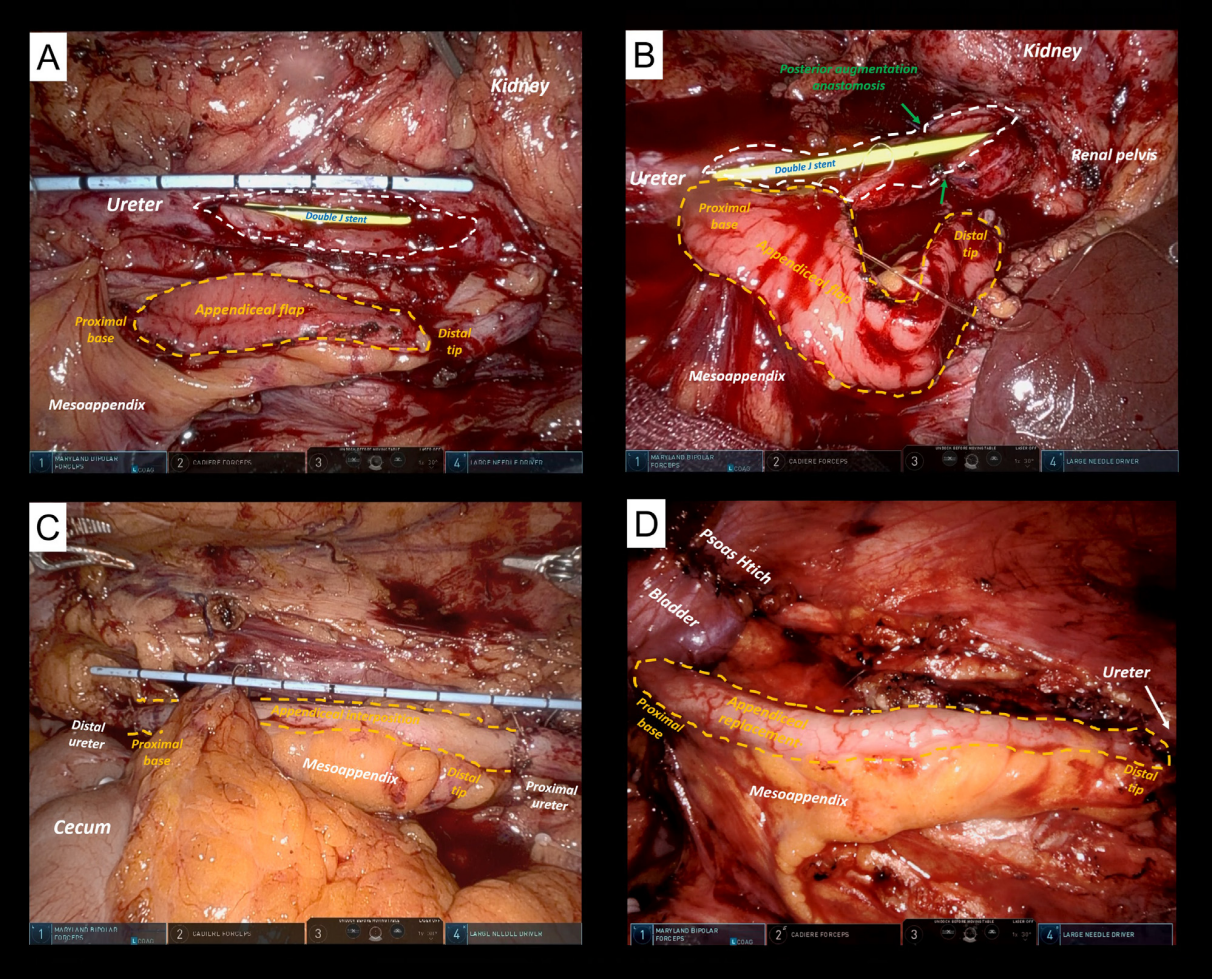
Intraoperative views of robotic appendiceal ureteroplasty. Yellow dotted line: appendix; and white dotted line: ureter. (A) Simple appendiceal flap: The detubularized appendiceal flap is anastomosed to the ureteral defect in an onlay fashion. (B) Appendiceal flap with posterior augmentation anastomosis: Green arrows indicate the sutures of posterior augmentation anastomosis. (C) Appendiceal interposition: The tubularized and isoperistaltic appendix is interposed between the proximal and the distal ureteral segments. (D) Appendiceal ureteral replacement with psoas hitch: The appendix is used to replace an extensive ureteral defect extending from the renal pelvis or the upper ureter to the bladder, while psoas hitch ensures a tension-free anastomosis.

For ureteral stricture without luminal obliteration, the ureter was longitudinally incised along the ventral side until a patent lumen was obtained (Fig. 2A). An 8-Fr catheter was routinely used to insert the lumen to confirm luminal continuity, after which the length of the ventral defect was measured. Afterwards, a double-J stent was placed by the antegrade method along a nitinol hydrophilic guide wire from the incision in the ureter. The appendix with the required length was harvested and spatulated along the antimesenteric border. Meticulous hemostasis of the split appendiceal edges should be achieved using robotic monopolar scissors with low-energy cautery before commencing the anastomosis, to prevent oozing and to ensure a clear operative field. The appendiceal flap was anastomosed to the ureteral defect in an onlay fashion using 4–0 barbed sutures. The appendix was generally oriented in an isoperistaltic direction, although this might not be considered mandatory for the use of the onlay flap technique. The edge of the appendiceal mesentery was then fixed to adjacent tissues with several interrupted sutures to reduce traction and tension (Fig. 2A).

##### 2.1.1.2 Appendiceal flap with posterior augmentation anastomosis

For ureteral strictures with short luminal obliteration or those requiring excision of short segmental lesions, the posterior augmentation anastomosis technique should be performed to rebuild a smooth posterior ureteral wall (Fig. 2B) [15]. Occasionally, this technique was also used to narrow the length of ureteral defect [15]. Subsequent steps were performed as previously described for the simple appendiceal flap technique (Fig. 2B).

#### 2.1.2 Appendiceal interposition

For ureteral stricture with long luminal obliteration, or those requiring excision of the long segmental lesions, the posterior augmentation anastomosis technique was not feasible; therefore, the appendiceal interposition technique was employed (Fig. 2C). The appendix with the required length was harvested, and the orientation was kept isoperistaltic. A 10-Fr catheter was inserted into the appendiceal lumen to remove fecaliths or other contents and to prevent subsequent obstruction. The appendix was spatulated ∼1 cm along the antimesenteric border at the tip end and along the mesenteric border at the base end to fit the ureteral ends. Posterior wall anastomosis was first performed in a continuous fashion at the two ends using 4–0 barbed sutures or 5–0 vicryl sutures, ensuring precise mucosa-to-mucosa alignment. Afterwards, a double-J stent was inserted from the proximal defect along a nitinol hydrophilic guide wire by the antegrade method. Finally, the anterior walls were closed with similar techniques (Fig. 2C). Gentle irrigation with saline water from nephrostomy confirmed luminal patency and watertight anastomosis.

#### 2.1.3 Appendiceal ureteral replacement

For long-segment ureteral strictures in which posterior augmentation anastomosis was not feasible and the distal ureter was inadequate for appendiceal interposition, appendiceal ureteral replacement might be needed (Fig. 2D).

The proximal ureter or renal pelvis was mobilized to obtain healthy tissue for anastomosis. A right-sided psoas hitch of the bladder was then performed to shorten the anastomotic distance. The distance between the proximal ureteral end or renal pelvic end and the bladder was routinely assessed to determine whether the available appendiceal length was sufficient for ureteral replacement. If the length of the appendix was inadequate, IUR was considered as an alternative option. If the length of the appendix was sufficient, the appendix was utilized for ureteral replacement.

The appendiceal harvest and preparation methods were the same as those used in the interposition technique. An isoperistaltic orientation must be maintained. The posterior wall of the proximal ureteroappendiceal or pyeloappendiceal anastomosis was completed first, followed by antegrade placement of a double-J stent through the proximal defect. Thereafter, the anterior wall of the proximal anastomosis was closed, and the base end of the appendix was implanted into the bladder to complete the appendiceovesical anastomosis (Fig. 2D). Alternatively, the posterior wall of the proximal anastomosis and one side of the appendiceovesical implantation could be completed first, followed by antegrade placement of a double-J stent and closure of the remaining anastomoses. All anastomoses were performed in a continuous, watertight fashion using 4–0 barbed sutures or 5–0 vicryl sutures.

Intravenous indocyanine green with the Firefly system was used as needed to evaluate the blood supply intraoperatively.

### 2.2 Postoperative management and follow-up

A liquid diet and water were provided on postoperative day (POD) 1. Intravenous antibiotics (cefotiam and metronidazole) were administered for 1–3 d postoperatively. The nephrostomy tube was closed on POD 7. The Foley catheter was removed on POD 7. The drainage tube was usually removed when the drained fluid was <50 ml, without concern about urine leakage. The double-J stent was removed 2–3 mo after surgery. Anterograde urography with the videourodynamic examination was performed 1 wk after stent removal to assess the patency of the anastomosis. If the ureter was patent, the nephrostomy tube was removed. Functional cine magnetic resonance urography was performed at 3 mo postoperatively, computed tomography urography was performed at 6 mo postoperatively, and ultrasound examinations were conducted every 3 mo. Diuretic renogram was normally performed at 6 mo postoperatively, but it was not mandatory.

The functional outcome was evaluated by delta estimated glomerular filtration rate (ΔeGFR), which was calculated by eGFR at the last follow-up and the eGFR before surgery using the formula ([post-eGFR–pre-eGFR]/pre-eGFR). Decreased renal function was defined as a ΔeGFR of <–0.10 ml/min/1.73 m^2^. Stable renal function was defined as a ΔeGFR of –0.10 to <0.10 ml/min/1.73 m^2^. Improved renal function was defined as a ΔeGFR of >0.10 ml/min/1.73 m^2^.

The criteria for complete success during the follow-up period were defined as the absence of clinical symptoms and no evidence of obstruction at the site of ureteral surgery on radiographic evaluation. Success rates were reported with 95% confidence intervals calculated using the Clopper-Pearson exact method.

## 3 Results

As shown in Table 1, 17 males and 14 females underwent RAU. The affected side in all the patients was the right side. The etiologies associated with ureteral strictures included failed pyeloplasty (6/31), iatrogenic ureteral stricture following ureteral lithotripsy (21/31), and idiopathic cause (4/31). Ureteral strictures were classified by location as ureteral pelvic junction (UPJ)–proximal (9/31), proximal (13/31), proximal-middle (3/31), middle (4/31), middle-distal (1/31), and panureteral (1/31). The degree of ureteral lumen obliteration was classified as a completely obliterated lumen (14/31) or a partially obliterated lumen (17/31). The mean stricture length was 5.5 cm (range, 3–25 cm).

**Table 1:** Patients’ characteristics and preoperative findings

Variable	Results
The median age (yr), range	38 (20–58)
Gender, *n* (%)	
Male	17 (54.8)
Female	14 (45.2)
The mean BMI (kg/m^2^), range	24.8 (16–38.2)
Presenting symptoms, *n* (%)	
Flank pain	27 (87.1)
Asymptomatic	4 (12.9)
Etiology associated with stricture, *n* (%)	
Failed pyeloplasty	6 (19.4)
Robotic	2 (6.5)
Laparoscopic	1 (3.2)
Open	3 (9.7)
Iatrogenic ureteral stricture following ureteral lithotripsy	21 (67.7)
Idiopathic	4 (12.9)
History of laparoscopic ureterolithotomy, *n* (%)	1 (3.2)
Prior ureteral balloon dilatation, *n* (%)	9 (29.0)
Stricture location, *n* (%)	
UPJ–proximal	9 (29.0)
Proximal	13 (41.9)
Proximal-middle	3 (9.7)
Middle	4 (12.9)
Middle-distal	1 (3.2)
Panureteral	1 (3.2)
Degree of ureteral lumen obliteration, *n* (%)	
Completely obliterated lumen	14 (45.2)
Partially obliterated lumen	17 (54.8)
The mean stricture length (cm), range	5.5 (3–25)
Simple appendiceal flap	4.5 (3–6)
Appendiceal flap with posterior augmentation anastomosis	4.3 (3–6)
Appendiceal interposition	6 (5–8)
Appendiceal ureteral replacement with psoas hitch	17.5 (10–25)

BMI = body mass index; UPJ = ureteral pelvic junction.

The surgical techniques included four types: simple appendiceal flap (16/31), appendiceal flap with posterior augmentation anastomosis (9/31), appendiceal interposition (4/31), and appendiceal ureteral replacement with a psoas hitch (2/31). The mean operative time was 176.4 min (range, 99–265 min), with a mean estimated blood loss of 42.7 ml (range, 10–200 ml). The mean postoperative hospitalization duration was 4.5 d (range, 3–12 d). Postoperative urinary tract infection occurred in 4 patients (12.9%, Clavien-Dindo [CD] grade II), and no CD grade III–V complications were observed. The median follow-up time was 28 mo (range, 12–67 mo). The mean eGFR remained stable, with no significant difference between the preoperative value and postoperative value at 3 mo, 6 mo, or 1 yr (*p* > 0.05). The ΔeGFR were −0.01, 0.04, and −0.04 0.10 ml/min/1.73 m^2^, respectively. During follow-up, three patients had mild but stable, asymptomatic hydronephrosis with stable renal function. One patient developed right-sided ureteral obstruction 3 yr after surgery. Ureteroscopy was performed, and it confirmed that there was no obstruction at the appendiceal reconstruction site. Instead, a new obstruction was identified distal to the reconstructed segment, which was caused by an ovarian tumor diagnosed by gynecologic oncologist. At the time of follow-up, the patient remained alive and continued to have regular double-J stent exchanges. As the obstruction was unrelated to the reconstructed segment, surgical failure was not considered. However, to avoid potential misleading, this case was excluded from the success analysis; therefore, the overall success rate was 100% (30/30), with a 95% confidence interval of 88.4–100% (Table 2).

**Table 2:** Intraoperative details and follow-up results

Variable	Results	
Surgical techniques of ureteral reconstruction, *n* (%)		
Simple appendiceal flap	16 (51.6)	
Appendiceal flap with posterior augmentation anastomosis	9 (29.0)	
Appendiceal interposition	4 (12.9)	
Appendiceal ureteral replacement with psoas hitch	2 (6.5)	
Mean operative time (min), range	176.4 (99–265)	
Simple appendiceal flap	153.9 (99–221)	
Appendiceal flap with posterior augmentation anastomosis	176.3 (139–211)	
Appendiceal interposition	229 (183–265)	
Appendiceal ureteral replacement with psoas hitch	251 (247–255)	
Mean estimated blood loss (ml), range	42.7 (10–200)	
Simple appendiceal flap	25.6 (10–100)	
Appendiceal flap with posterior augmentation anastomosis	53.3 (10–200)	
Appendiceal interposition	53.8 (30–100)	
Appendiceal ureteral replacement with psoas hitch	110 (20–200)	
Mean postoperative hospitalization (d), range	4.5 (3–12)	
Postoperative complications, CD grade, *n* (%)		
CD grade Ⅰ–Ⅱ		
UTI (grade Ⅱ)	4 (12.9)	
CD grade Ⅲ–Ⅴ	0	
Mean eGFR by CKD-EPI creatinine equation (ml/min/1.73 m^2^)		
Preoperative 1-d	90.9	
		***p* value**
Postoperative 3-mo	84.3	0.30
Postoperative 6-mo	91.1	0.98
Postoperative 1-yr	92.2	0.84
Median follow-up time (mo), range	28 (12–67)	
The overall success rate (%)	100 (30/30)	

CD = Clavien-Dindo; CKD-EPI = Chronic Kidney Disease Epidemiology Collaboration; eGFR = estimated glomerular filtration rate; UTI = urinary tract infection.

## 4 Discussion

Here, we report the midterm outcomes of RAU for the management of complex ureteral strictures. To our knowledge, this is the largest single-center cohort of patients who underwent RAU. All surgeries were performed safely without severe intraoperative or postoperative complications. All patients achieved successful reconstruction during follow-up. These results suggest that RAU can serve as a safe and reliable option for right-sided ureteral reconstruction.

More than a century has passed since Melnikoff first reported the use of the appendix for ureteral substitution in 1912 [9]. Many reports have shown that the appendix can be used for various purposes to repair ureteral strictures or defects in both adults [7], [8], [12], [14] and children [16], [17], [18]. In 2009, Reggio et al. [13] reported the first laparoscopic appendiceal flap ureteroplasty, which demonstrated favorable outcomes. Duty et al. [12] reported six cases of robotic appendiceal onlay flap ureteroplasty, achieving a 100% objective success rate in 2015. In 2017, Yarlagadda et al. [19] reported the first robotic appendiceal interposition ureteroplasty to successfully repair a 5 cm right-sided obliterative ureteral stricture, with an operation duration of 379 min.

With the evolution of robotic surgery, compared with open and conventional laparoscopic approaches, robotic ureteral reconstruction has shown significant advantages. Robotic ureteral reconstruction offers increased dexterity, improved visualization, reduced blood loss, shorter hospital stays than open approaches, as well as shorter operative times than laparoscopic approaches [20]. These benefits expand the reconstructive options available for complex ureteral strictures. Although high-level comparative data are lacking, we believe that robotic surgery can help achieve superior reconstructive accuracy and, ultimately, better clinical outcomes. Nevertheless, most published reports on AU have involved open or laparoscopic procedures, whereas reports concerning RAU remain limited. In our early experience, five cases of laparoscopic appendiceal onlay flap ureteroplasty were performed [7]. Following the adoption of the robotic system, nearly all subsequent cases were completed robotically with satisfying results [7], [8].

The AU provides several distinct advantages. The blood supply of the appendix is good, and the appendix can be harvested easily. The avoidance of bowel anastomosis can reduce the risk of intestinal leakage and allow early resumption of oral intake [2]. Moreover, the relatively small mucosal surface area of the appendix minimizes the risk of metabolic acidosis. However, despite its advantages, the AU has inherent limitations, including prior appendectomy, severe appendiceal inflammation, a narrow appendiceal lumen, and inadequate length. Therefore, careful patient selection is necessary [2]. Since the suitability of the appendix for ureteral reconstruction can only be determined intraoperatively, alternative strategies should be prepared in advance. If the appendix is unsuitable, OMG ureteroplasty or IUR should be considered, which requires surgeons to be skilled in multiple surgical techniques.

**Fig. 3:**
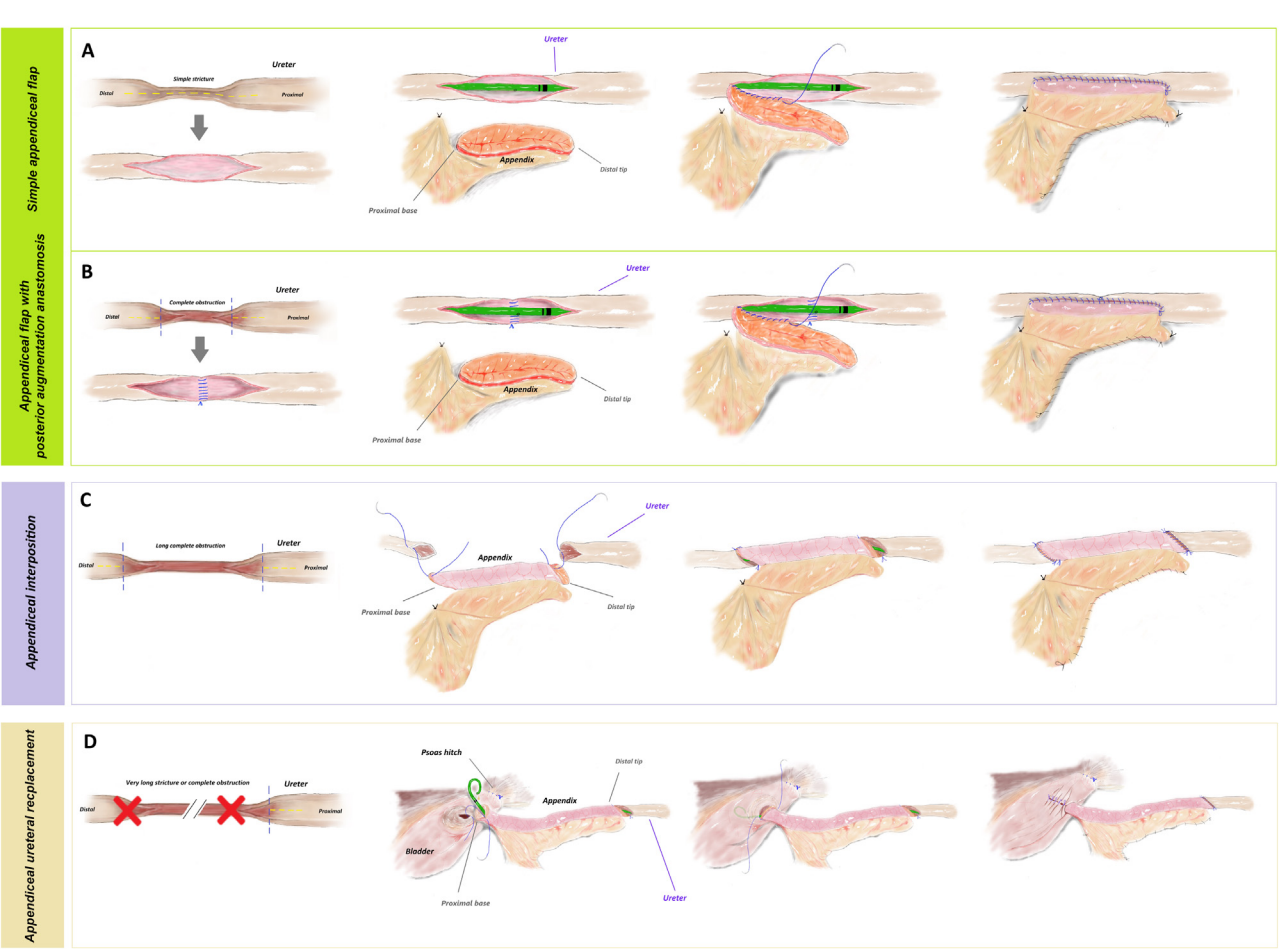
Schematic illustrations of robotic appendiceal ureteroplasty. (A) Simple appendiceal flap: Indicated for ureteral strictures without luminal obliteration. (B) Appendiceal flap with posterior augmentation anastomosis: Indicated for ureteral strictures with short luminal obliteration or those requiring excision of short segmental lesions. (C) Appendiceal interposition: Indicated for ureteral strictures with long luminal obliteration, or those requiring excision of the long segmental lesions when the posterior augmentation anastomosis is not feasible. (D) Appendiceal ureteral replacement with psoas hitch: Indicated for long-segment ureteral strictures in which posterior augmentation anastomosis is not feasible and the distal ureter is inadequate for appendiceal interposition.

**Supplementary Figure 1:**
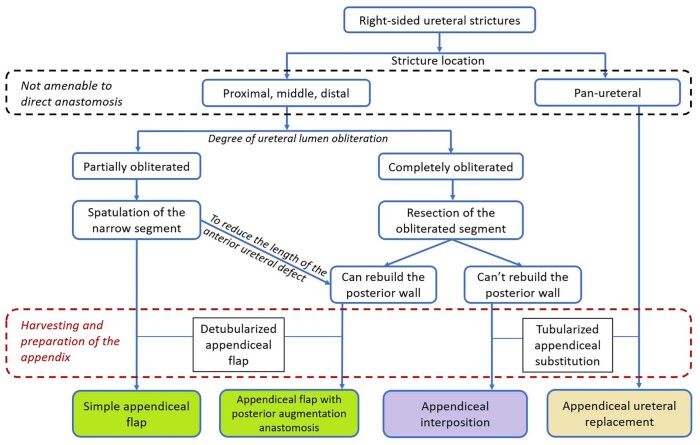
Flowchart of decision-making for appendiceal ureteral reconstruction in right-sided ureteral strictures.

Ureteral reconstruction techniques can be broadly categorized into three main strategies: reduction of the distance to achieve tension-free anastomosis, expansion of the ureteral lumen, and ureteral substitution or renal autotransplantation. The appendix can be used to repair the ureter in either a detubularized onlay flap or a tubularized substitution manner, depending on whether the ureteral stricture is partially obliterated or completely obstructed, whether segmental resection is needed, and whether posterior augmentation anastomosis is feasible, and on the overall length of the diseased ureter (Fig. 3). The definitive decision regarding its use will be made intraoperatively, as the applicability of the appendix depends on its length and quality, which can only be accurately assessed during surgery. The decision-making process is shown in the Supplementary Figure 1. Compared with OMG, the use of a detubularized flap provides the unique advantage of an intrinsic blood supply, ensuring reliable vascularization and healing. When it is used in a tubularized fashion, its small luminal diameter closely matches that of the ureter, allowing for direct anastomosis at both ends without the need for additional tailoring or suturing procedures as required in reconfigured Yang-Monti ileal or colonic reconstructions [4]. Since the appendix is part of the intestine, its mucosa secretes mucus. When using it, care must be taken to thoroughly clear any debris from the lumen, and follow-up should monitor potential mucus obstruction. To date, no patient has experienced mucus-related blockage.

In our experience, when the ureteral defect is very short (eg, <2 cm), using the appendix either as an onlay flap or as an interposition is not recommended as the first choice because of the heightened challenge of anastomosis and increased risk of stricture recurrence. In such cases, OMG may serve as an alternative strategy. For proximal ureteral or UPJ reconstruction, extensive mobilization of the ileocecal region is essential to reduce tension on the appendiceal mesentery before tension-free anastomosis can be achieved.

Our study inevitably has several limitations. This was a single-center cohort study with a relatively small number of patients, and longer follow-up is needed to confirm the long-term outcomes. In addition, all included cases involved right-sided ureteral strictures. The four appendiceal reconstructive techniques were analyzed together, and the number of cases for some individual techniques was relatively small. Therefore, meaningful subgroup comparisons could not be performed. Future studies with larger sample sizes and longer follow-up time are required to enable stratified analyses and to compare the outcomes of different appendiceal techniques. In the literature, several case reports have described the use of the appendix for left-sided ureteral reconstruction, mainly via an open approach [6], [16], [21], [22] and rarely through the use of robotic or laparoscopic approaches [17], [18], [23]. Left-sided appendiceal ureteral reconstruction in adults is technically demanding. The extensive mobilization and transposition of the ileocecal region may increase the risk of internal hernia and is challenging to achieve under a robotic single-docking setup [22], [24], [25]. Therefore, the majority of left-sided similar cases in our practice have been managed with OMG ureteroplasty with favorable outcomes. In future research, we will carefully select left-sided candidates for AU. Another limitation of this study is the use of ΔeGFR, as compensatory hyperfunction of the contralateral kidney can preserve total eGFR, despite functional decline in the operated unit. Therefore, eGFR changes should be interpreted with caution. We also agree that more comprehensive functional assessment, such as differential renal function by renal scan, may provide additional accuracy, but was not uniformly available in all patients during follow-up.

## 5 Conclusions

Our single-center study demonstrated that RAU, performed using the onlay flap, interposition, or total replacement techniques, can be safely and effectively applied for complex right-sided ureteral strictures, with low complication rates and high success rates. However, multicenter, large-sample, and long-term studies are still needed to confirm these findings.

  ***Author contributions***: Kunlin Yang and Xuesong Li had full access to all the data in the study and takes responsibility for the integrity of the data and the accuracy of the data analysis.

  *Study concept and design*: Yang, Li.

*Acquisition of data*: Yang, Huang, Zhu, Li.

*Analysis and interpretation of data*: Yang, Li, Xu.

*Drafting of the manuscript*: Yang.

*Critical revision of the manuscript for important intellectual content*: Yang.

*Statistical analysis*: Yang.

*Obtaining funding*: Yang.

*Administrative, technical, or material support*: Yang, Zhou, Li.

*Supervision*: Yang, Zhou, Li.

*Other* (specify): None.

  ***Financial disclosures:*** Kunlin Yang and Xuesong Li certify that all conflicts of interest, including specific financial interests and relationships and affiliations relevant to the subject matter or materials discussed in the manuscript (eg, employment/affiliation, grants or funding, consultancies, honoraria, stock ownership or options, expert testimony, royalties, or patents filed, received, or pending), are the following: None.

  ***Funding/Support and role of the sponsor*:** This study was supported by the National Natural Foundation of China (No. 82400787) and the Beijing Nova Program (No. 20250484918).

  ***Declaration of generative AI and AI-assisted technologies in the writing process*:** During the preparation of this manuscript, the authors used ChatGPT solely to assist with language editing of the manuscript to enhance the English expression. After using this tool, the authors subsequently reviewed and edited the content as needed. In addition, the authors invited native English speakers to review the language again to ensure its accuracy. The authors take full responsibility for the content of the publication.
